# Cardiovascular functions and arterial stiffness after JUUL use

**DOI:** 10.18332/tid/144317

**Published:** 2022-04-01

**Authors:** Solveig Gernun, Klaas F. Franzen, Nadja Mallock, Julia Benthien, Andreas Luch, Kai Mortensen, Daniel Drömann, Oliver Pogarell, Tobias Rüther, Andrea Rabenstein

**Affiliations:** 1 Department of Psychiatry and Psychotherapy, Klinikum der Universität München, Munich, Germany; 2 Medizinische Klinik III, Universitätsklinikum Schleswig-Holstein, Lübeck, Germany; 3 Airway Research Center North, Germany; 4 Department of Chemical and Product Safety, German Federal Institute for Risk Assessment, Berlin, Germany; 5 Institute of Pharmacy, Department of Biology, Chemistry and Pharmacy, Freie Universität Berlin, Berlin, Germany; 6 Cardiology Kiel, Kiel, Germany

**Keywords:** arterial stiffness, smoking, JUUL, electronic cigarette, cigarette

## Abstract

**INTRODUCTION:**

The rapid growth in the e-cigarette market after the launch of JUUL e-cigarettes led to much discussion on the potential benefits and risks of pods, JUUL devices, and conventional e-cigarettes compared with combustible cigarettes. Independent data are required to assess the effects of these products on cardiovascular surrogate parameters and cardiovascular risk.

**METHODS:**

We conducted a single-center three-arm study comparing combustible cigarettes with JUUL e-cigarettes with the old and new technology. We recruited 32 participants who were active smokers (n=15) or vapers (n=17) and performed a total of 39 measurements before and 5, 15, and 30 minutes, after participants smoked a combustible cigarette or vaped a JUUL e-cigarette with the new or old technology. Measurements included peripheral and central blood pressures and parameters of arterial stiffness, including pulse wave velocity and augmentation index.

**RESULTS:**

Peripheral systolic blood pressure, central blood pressure, and peripheral pulse rate increased significantly in all three groups (each p<0.05). Heart rate (HR) changes lasted significantly longer than blood pressure changes. The augmentation index and pulse wave velocity increased in all three groups, and a multivariate analysis of variance showed that the increases were independent of systolic blood pressure, sex, age, device, and HR.

**CONCLUSIONS:**

Changes in blood pressure and arterial stiffness are similar after cigarette smoking and JUUL use. These changes may be associated with an increased cardiovascular risk compared with no product use. However, a long-term follow-up evaluation of JUUL use and a head-to-head comparison with conventional e-cigarettes are still needed.

## INTRODUCTION

After the launch of the JUUL device on the US market in 2015, retail sales increased over the following three years, and the device became the best selling e-cigarette with 40% of the retail market share^1^. As a result of marketing and advertising strategies, JUUL became the e-cigarette brand used by over half of middle and high school students^2^, and its widespread use among young people led to much discussion in the entire field of healthcare providers, not just among pediatricians. Besides advertising, this discussion referred to the lack of information about the health risks that may be associated with e-cigarettes and other nicotine delivery devices^3^. As a result of the popularity of e-cigarettes among young people and the outbreak of e-cigarette or vaping product use-associated lung injury (EVALI) in October 2019^4^, the FDA passed a legislative decision in January 2020 that tobacco products may be sold only to people aged ≥21 years^5^.

The large market share of e-cigarettes and their popularity among young people in the US increased interest not only in the question of long-term harm but also the gateway hypothesis^6^. Studies have shown that vaping may increase the risk of initiation of cigarettes, especially among teenagers^7,8^. Smoking is still one of the leading causes of mortality in Germany and is responsible for 110000–140000 premature deaths^9^. The levels of many hazardous and potentially hazardous substances are significantly lower in e-cigarette emissions than in cigarette smoke, leading to a reduced exposure to these compounds while vaping^10,11^. Thus, e-cigarette use is expected to reduce the risks for some diseases, especially cancer, and therefore JUUL and other kinds of e-cigarettes have started to be used as tools for smoking cessation^12^. However, e-cigarettes still deliver nicotine, so their use is less likely to reduce the rate of cardiovascular diseases, although this aspect requires further research.

In general, smoking has been described as one of the most important risk factors for cardiovascular events^13,14^, but it is also one of the most modifiable risk factors^15^. Several studies evaluated the chronic effects of smoking on cardiovascular events. To assess subclinical end-organ damage, the European Society of Hypertensiology and European Society of Cardiology recommend measuring arterial stiffness and endothelial dysfunction, parameters that also provide some initial indications of the onset of systemic cardiovascular disease^16^. A clear association between smoking and the increase in arterial stiffness is known^15,17^ and several studies have shown the rise in arterial stiffness of both e-cigarettes and heated tobacco products^18-20^. Some evidence shows that e-cigarettes, which are often marketed as a healthier smoking alternative^21^, can indeed increase cardiovascular risks. For example, Alzahrani et al.^22^ found that non-daily and in particular daily e-cigarette use increased the risk of myocardial infarction. However, a recent epidemiological analysis did not confirm increased cardiovascular risks for vapers who never smoked cigarettes^23^. Because conclusive evidence is still limited^24^, more data and analyses are required, especially for devices that deliver high levels of nicotine, such as the JUUL^25^. Although JUUL pods sold in the US contain up to 58 mg/mL of nicotine, the maximum concentration of nicotine in e-liquids allowed in the European Union is 20 mg/mL^26^. Therefore, in the European Union conventional JUUL pods were replaced by what JUUL lab employees supposedly refer to as ‘Turbo JUUL’ which uses different wick material^27^. A study on the Turbo JUUL found that the new wick material increased the amount of vapor generated and subsequently increased the amount of nicotine released per puff^25^.

To date, information is lacking on the effects of JUUL use on the pathogenesis of cardiovascular diseases, specifically on arterial stiffness and endothelial dysfunction. Therefore, we aimed to compare the acute effects on peripheral and central blood pressure and arterial stiffness of the new and old JUUL devices and conventional cigarettes, assuming that the use of the European JUUL versions as well as cigarette consumption increases the respective parameters.

## METHODS

### Study cohort and design

This single-center, three-arm study included 15 active smokers and 17 active e-cigarette users. A total of 39 measurements were performed, whereby the e-cigarette users were mostly tested in both the old and the new JUUL groups in order not to offer an entry into ‘dual use’ which seems to be a possible potentiation of health risks^28^. Participants were recruited from the population of the city of Munich by advertising and were assigned to the tobacco cigarette group or one of the two JUUL groups (old and new JUUL technology) according to the product that they normally used. At screening, inclusion criteria were checked as follows: 1) smoker or vaper, 2) no mental disorder, 3) no cardiovascular disease, 4) no thyroid disease, 5) no diabetes, 6) no abnormalities in physical examination, 7) no hypertension, and 8) no hypercholesterolemia. In addition, a pregnancy test was performed to exclude possible pregnancies in women who were not taking oral contraception. The measurements were made during the same phase of the menstrual cycle or pill period. All participants were asked to follow the guidelines for measuring arterial stiffness^29,30^. Therefore, cigarette smoking and nicotine intake in general were prohibited for the 12 hours before the study visit. The smoke-free time was tested by a Micro+Smokerlyzer™ (Bedfont Scientific Ltd., England) with a cut-off of 5 ppm carbon monoxide. In e-cigarette users, concentrations in the baseline blood sample were checked for possible elevated nicotine concentration. Participants were excluded if they declared that they were strict non-smokers. All participants provided written informed consent. The study was approved by the ethics committee of the LMU Munich (Amendment to project number 72-15) and performed in accordance with the principles of the Declaration of Helsinki in the currently valid version and was registered at the German Clinical Trials Register (DRKS, www.drks. de, registration number DRKS00017432). It was conducted according to the World Medical Association Declaration of Helsinki.

The three different study arms used the following products: 1) a commercial, combustible tobacco cigarette (Cig group; Marlboro Red, Philip & Morris, 0.8 mg nicotine); 2) a JUUL e-cigarette with the new technology (new JUUL group); and 3) a JUUL e-cigarette with the old technology (old JUUL group). The use of the JUUL device was explained to participants in groups 2) and 3) on the basis of the producer’s manual and vapers who were inexperienced in the use of JUUL e-cigarettes were trained in their use before the study. JUUL Pods with the flavor ‘Rich Tobacco’ were used and vegetable glycerol (VG), propylene glycol (PG), nicotine, benzoic acid and ‘aromas’ are specified as ingredients by the manufactor. Both the old and the new JUUL pods do not seem to differ much in the composition of ingredients. The VG/PG ratio is approximately 2:1 (w/w)^25^. All participants had to consume the respective product depending on the group affiliation at a frequency of 1 puff every 30 s for 10 puffs, with a puff duration of 3 s, as described in other publications^31^.

During the consumption sessions in the tobacco cigarette and new/modified JUUL arms, electroencephalography (EEG) measurements and blood samples for nicotine concentration during and after consumption were taken. Results on EEG and nicotine concentration in venous blood will be analyzed and published separately.

Generally, measurements were started at least 15 minutes before smoking or JUUL use. Peripheral and central hemodynamics were measured by the Mobil-O-Graph™ (I.E.M., Stollberg, Germany)^32-34^ before and at three times after using the specific device: at 5 minutes, 15 minutes, and at 30 minutes.

### Measurement of peripheral and central blood pressures and arterial stiffness

Blood pressure and additional variables were measured with the validated Mobil-O-Graph™ (software version HMS CS 4.2, I.E.M. GmbH), which allows peripheral and central blood pressures, heart rate and arterial stiffness to be recorded^32,33,35^. Like many other devices, the Mobil-O-Graph™ uses the oscillometric technique in which a standard blood pressure cuff is placed at the arteria brachialis^32^. Besides the peripheral blood pressure, heart rate is also measured using the oscillometric technique. Central systolic blood pressure is determined from brachial waveforms which are recorded with the cuff at the level of diastolic blood pressure and processed with the ARCSolver transfer function. In addition, stroke volume and total peripheral resistance are calculated via a transfer function similar to the ARCSolver transfer function^32^. The derived central waveforms are used for pulse waveform analysis and the augmentation index and augmentation pressure are derived from them^33,34^. The augmentation index is adjusted for a heart rate of 75 beats per minute (bpm). After participants had rested for a short period, measurements were made at least 15 minutes before participants smoked or vaped in a sitting position. These data were used as a baseline and as references for statistical analyses.

### Statistical analysis

Statistical analyses and graph editing were performed with SPSS statistical software (SPSS 23 Inc., Chicago, IL, USA) and GraphPad Prism 4 (GraphPad Software Inc., San Diego, CA, USA), respectively. Baseline mean values of blood pressure and arterial stiffness were used as statistical references.

Before further analysis, peripheral and central blood pressures were analyzed for normal distribution by Kolmogorov-Smirnov tests. Because of the longitudinal design, we used a two-way repeated-measures analysis of variance (ANOVA) to evaluate the effect of time. Accordingly, if we found no interaction we performed *post hoc* tests (Bonferroni) with G*Power. Also, we analyzed the data with a paired Student’s t-test or Wilcoxon rank sum test, as appropriate, to compare measurements at the four times. Where applicable, we performed a multivariate analysis of variance (MANOVA) correcting for age, mean arterial pressure (MAP), heart rate (HR), and sex. If not otherwise stated, all data are expressed as mean ± SD. A p<0.05 was considered statistically significant.

## RESULTS

### Baseline characteristics

**Figure 1:**
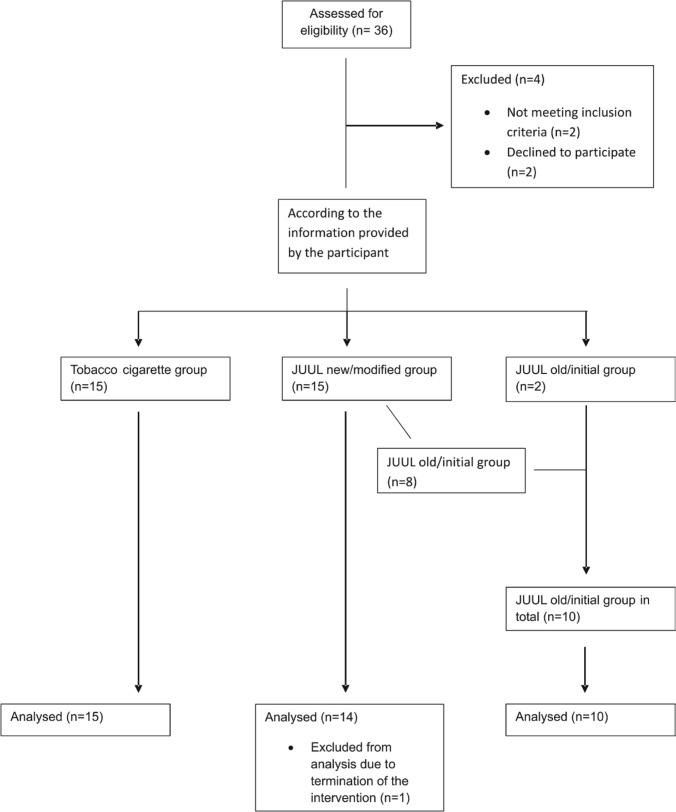
Consort flow chart of participants

**Figure 2:**
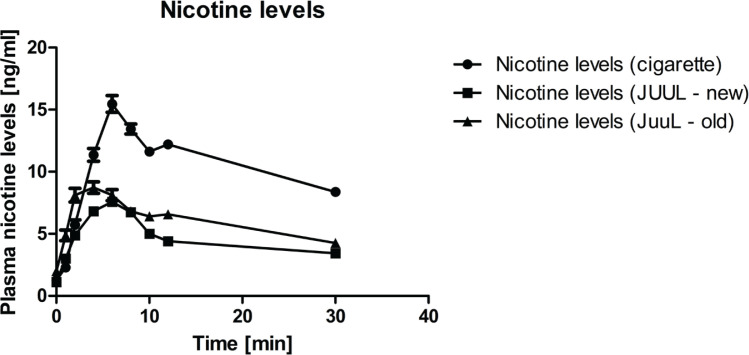
Plasma nicotine levels. Data are expressed as mean (SEM)

Baseline characteristics for all 32 participants are presented in Table 1 and the flow of participants through the study is shown in Figure 1. The nicotine levels for the three groups with the corresponding numbers of participants are shown in Figure 2 ^36^.

**Table 1:** Baseline characteristics of participants (N=39)

*Characteristics*	*Mean ± SD*
**Sex**, n	
Male	23
Female	16
**Age** (years)	32 ± 9.8
median (25% percentile; 75% percentile)	29 (25; 39)
**Height** (m)	1.75 ± 0.08
**Weight** (kg)	72.9 ± 11.1
**BMI** (kg/m²)	23.7 ± 3.3

### Peripheral systolic blood pressure and peripheral pulse pressure

**Figure 3:**
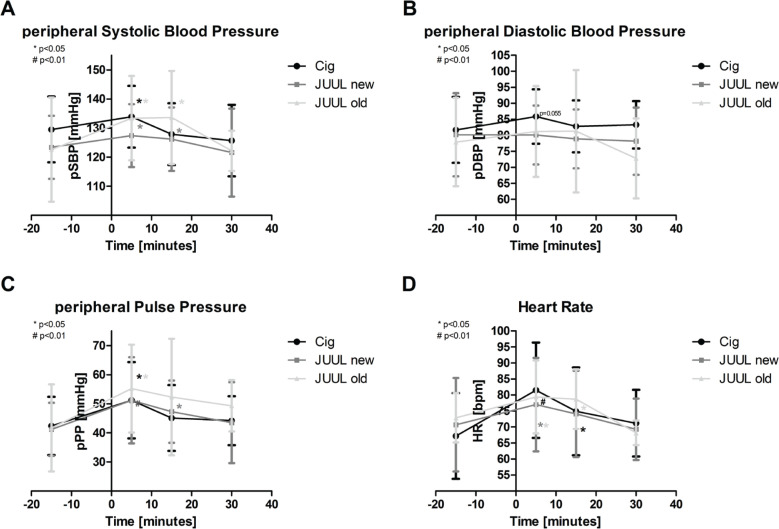
a) Peripheral systolic blood pressure (pSBP), b) peripheral diastolic blood pressure (pDBP), c) peripheral pulse pressure (pPP), and d) heart rate (HR). Asterisks indicate a significant change of individual values from baseline. Data are expressed as mean (SD)

At the initial measurement after 5 minutes, peripheral systolic blood pressure (pSBP) increased significantly by more than 3% in all three groups (p<0.05; Figure 3A, Table 2). In the JUUL new (+2%) and JUUL old groups (+9%), it remained elevated after 15 minutes (p<0.05; Figure 3A, Table 2).

**Table 2:** Effects of cigarette smoking and JUUL use on cardiovascular parameters

*Measurements*	*Cigarette (n=15)*						
	*Baseline*	*5 Minutes*	*p*	*15 Minutes*	*p*	*30 Minutes*	*p*
	*Mean ± SD*	*Mean ± SD*		*Mean ± SD*		*Mean ± SD*	
**pSBP** (mmHg)	129.5 ± 11.3	133.9 ± 10.6	0.0179	127.9 ± 10.6	0.4744	125.7 ± 12.3	0.0843
**pDBP** (mmHg)	81.7 ± 10.3	85.9 ± 8.5	0.0551	82.8 ± 8.1	0.6690	83.3 ± 7.4	0.4406
**pMAD** (mmHg)	103.1 ± 10.6	107.5 ± 8.1	0.0326	103.7 ± 7.5	0.6882	102.7 ± 8.3	0.7757
**pPP** (mmHg)	42.4 ± 10.0	51.2 ± 13.1	0.0342	45.1 ± 11.3	0.3685	44.2 ± 8.4	0.5757
**HR** (bpm)	67.2 ± 13.4	81.5 ± 14.9	0.0002	74.9 ± 13.7	0.0287	71.2 ± 10.4	0.1375
**cSBP** (mmHg)	113.1 ± 14.4	119.9 ± 11.1	0.0261	115.1 ± 10.9	0.3683	112.7 ± 11.4	0.8797
**cDBP** (mmHg)	83.0 ± 10.3	88.5 ± 7.9	0.0039	84.4 ± 7.5	0.5093	84.7 ± 7.6	0.3126
**cPP** (mmHg)	27.1 ± 8.8	34.2 ± 9.0	0.0149	30.8 ± 8.2	0.0985	28.1 ± 8.1	0.7182
**AIx@75** (%)	9.3 ± 10.4	18.4 ± 10.8	0.0003	14.5 ± 11.5	0.0689	9.5 ± 9.5	0.9517
**PWV** (m/s)	5.4 ± 0.5	5.7 ± 0.4	0.0287	5.5 ± 0.4	0.1140	5.4 ± 0.4	0.9484
**SV** (mL)	65.3 ± 12.4	75.5 ± 15.0	0.0136	75.6 ± 16.6	0.0104	73.2 ± 14.3	0.0940
**TPR** (mmHg×min/L)	1.2 ± 0.2	1.3 ± 0.2	0.0035	1.2 ± 0.2	0.2711	1.2 ± 0.2	0.7744
***Measurements***	***JUUL new technology (n=15)***						
	***Baseline***	***5 Minutes***	***p***	***15 Minutes***	***p***	***30 Minutes***	***p***
	***Mean ± SD***	***Mean ± SD***		***Mean ± SD***		***Mean ± SD***	
**pSBP** (mmHg)	123.4 ± 10.8	127.4 ± 10.8	0.0181	126.2 ± 10.9	0.0455	121.6 ± 15.1	0.8312
**pDBP** (mmHg)	80.2 ± 13.0	80.1 ± 9.2	0.1412	78.9 ± 9.2	0.3654	78.2 ± 10.5	0.6455
**pMAD** (mmHg)	99.4 ± 10.5	101.6 ± 8.4	0.0086	100.6 ± 8.4	0.0351	98.2 ± 10.9	0.5725
**pPP** (mmHg)	41.2 ± 9.1	51.2 ± 14.8	0.0084	47.3 ± 10.7	0.0290	43.5 ± 13.9	0.5989
**HR** (bpm)	70.7 ± 14.6	77.0 ± 14.6	0.0139	74.1 ± 13.5	0.2086	69.3 ± 9.6	0.9500
**cSBP** (mmHg)	113.2 ± 11.2	115.2 ± 8.7	0.0096	115.7 ± 7.5	0.0076	112.6 ± 13.6	0.4840
**cDBP** (mmHg)	82.4 ± 13.0	83.3 ± 8.8	0.0087	80.2 ± 9.4	0.6646	79.6 ± 10.6	0.9111
**cPP** (mmHg)	29.2 ± 6.1	34.3 ± 11.4	0.0755	35.2 ± 9.3	0.0055	33.2 ± 10.9	0.1242
**AIx@75** (%)	13.7 ± 12.3	16.1 ± 10.6	0.0045	13.3 ± 11.3	0.2350	11.0 ± 8.8	0.1157
**PWV** (m/s)	5.9 ± 1.1	5.9 ± 0.9	0.0088	6.0 ± 0.8	0.0140	5.9 ± 1.0	0.6367
**SV** (mL)	66.4 ± 14.3	76.8 ± 16.5	0.0052	80.3 ± 19.2	0.0120	77.6 ± 20.1	0.0606
**TPR** (mmHg×min/L)	1.2 ± 0.2	1.3 ± 0.1	0.0057	1.1 ± 0.2	0.2347	1.2 ± 0.3	0.4350
***Measurements***	***JUU L old technology (n=15)***						
	***Baseline***	***5 Minutes***	***p***	***15 Minutes***	***p***	***30 Minutes***	***p***
	***Mean ± SD***	***Mean ± SD***		***Mean ± SD***		***Mean ± SD***	
**pSBP** (mmHg)	122.6 ± 17.9	133.4 ± 14.5	0.0292	133.6 ± 16.0	0.0473	122.2 ± 6.9	0.9130
**pDBP** (mmHg)	77.9 ± 13.8	81.2 ± 14.2	0.1628	81.3 ± 19.1	0.4364	72.8 ± 12.5	0.9710
**pMAD** (mmHg)	99.5 ± 14.1	104.0 ± 11.8	0.0687	105.2 ± 14.8	0.0292	95.3 ± 9.5	0.7214
**pPP** (mmHg)	41.7 ± 15.0	55.2 ± 15.1	0.0125	52.3 ± 20.0	0.1911	49.3 ± 8.8	0.8258
**HR** (bpm)	72.9 ± 7.7	79.4 ± 11.4	0.0346	78.7 ± 9.3	0.0471	68.2 ± 3.8	0.3653
**cSBP** (mmHg)	111.8 ± 16.0	119.9 ± 15.5	0.0734	119.4 ± 16.0	0.0291	113.4 ± 6.9	0.8142
**cDBP** (mmHg)	79.3 ± 14.2	85.3 ± 14.5	0.0061	89.0 ± 17.9	0.0373	75.2 ± 13.4	0.9668
**cPP** (mmHg)	30.5 ± 10.9	36.9 ± 12.4	0.1091	32.7 ± 9.1	0.7259	30.0 ± 6.1	0.4632
**AIx@75** (%)	11.6 ± 4.3	23.5 ± 5.7	0.0011	21.6 ± 7.9	0.0020	17.8 ± 17.5	0.4229
**PWV** (m/s)	5.8 ± 1.1	6.0 ± 1.0	0.0349	5.8 ± 1.0	0.0467	5.8 ± 0.8	0.9423
**SV** (mL)	64.1 ± 16.0	70.4 ± 8.6	0.0058	69.9 ± 19.6	0.0857	65.7 ± 4.1	0.3907
**TPR** (mmHg×min/L)	1.2 ± 0.2	1.3 ± 0.2	0.1334	1.4 ± 0.2	0.0230	1.2 ± 0.3	0.8541

pDBP did not change significantly in either cigarette (Cig) or the old JUUL group (p>0.05; Figure 3B, Table 2) or the new JUUL group (p>0.05; Figure 3B, Table 2).

Peripheral mean arterial pressure (pMAP) increased significantly in the Cig (+4%) and new JUUL (+2%) groups after 5 minutes, and in the new JUUL (+ 1%) and old JUUL (+5%) groups after 15 minutes (each p<0.05; Table 2).

Peripheral pulse pressure (pPP) increased significantly, by more than 20%, in all three study groups after 5 minutes (p<0.05; Figure 3C, Table 2) and by 14% in the new JUUL group after 15 minutes (p<0.05; Figure 3C, Table 2).

### HR

After 5 minutes, HR increased significantly, by more than 8%, in all three study groups (each p<0.05; Figure 3D, Table 2). HR remained significantly increased after 15 minutes in the Cig and old JUUL groups (each p<0.05; Figure 3D, Table 2).

### Central blood pressure

**Figure 4:**
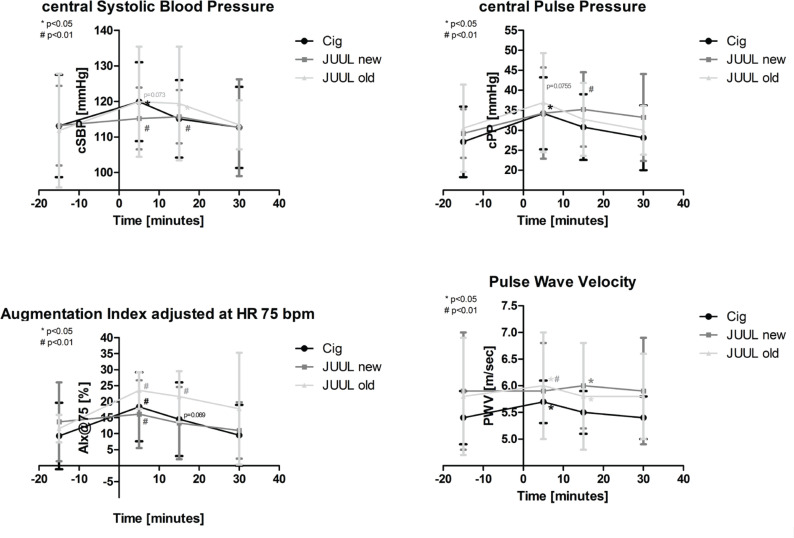
a) Central systolic blood pressure (cSBP), b) central pulse pressure (cPP), and, as measures of arterial stiffness, c) augmentation index adjusted for a heart rate of 75 beats per minute (AIx@75) and d) pulse wave velocity (PWV). Asterisks indicate a significant change of individual values from baseline. Data are expressed as mean (SD)

After the first 5 minutes, central systolic blood pressure (cSBP) increased significantly, by more than 6%, in the Cig group (p<0.05; Figure 3A, Table 2) and more than 1% in the new JUUL group (p<0.05; Figure 4A, Table 2). After 15 minutes, cSBP remained elevated in both JUUL groups (new JUUL +2%; old JUUL +6%; each p<0.05; Figure 4A, Table 2).

Central diastolic blood pressure (cDBP) was significantly increased after 5 minutes in the Cig (+6%), new JUUL (+1%), and old JUUL (+7%) groups (each p<0.01; Table 2), and after 15 minutes remained elevated in the old JUUL group (+12%; p<0.05; Table 2).

Central pulse pressure (cPP) increased significantly, by more than 20%, in the Cig group after 5 minutes and by more than 20% in the new JUUL group after 15 minutes (each p<0.05; Figure 4B, Table 2).

### Arterial stiffness and total peripheral resistance

The augmentation index adjusted for a heart rate of 75 bpm (AIx@75) increased significantly in the Cig (+97%), new JUUL (+17%), and old JUUL groups (+102%) after 5 minutes and in the old JUUL group (+86%) after 15 minutes (each p<0.01; Figure 4C, Table 2). After performing a multivariate analysis of AIx@75 including blood pressure and heart rate, in all three groups changes remained significant (Box test, each p>0.05; Levene test, each p<0.05; Wilks’ lambda [multivariate test], each p<0.05).

The pulse wave velocity (PWV) showed a significant change after 5 minutes in the Cig (+5%), new JUUL (+1%), and old JUUL (+2%) groups and after 15 minutes in the new JUUL (+1%) and old JUUL (+1%) groups (each p<0.05; Figure 4D, Table 2).

Stroke volume (SV) increased significantly, by more than 15%, in the Cig group, by more than 15% in the new JUUL group, and by more than 9% in the old JUUL group after 5 minutes, and by more than 15% in the Cig and by more than 20% in the new JUUL groups after 15 minutes (each p<0.05; Table 2).

Total peripheral resistance (TPR) increased after 5 minutes in the Cig (+11%) and new JUUL (+4%) groups and after 15 minutes in the old JUUL group (+16%) (each p<0.05; Table 2).

## DISCUSSION

To the best of our knowledge, this study is one of the first to compare the impact of vaping the European JUUL version and cigarette smoking on peripheral and central hemodynamics and arterial stiffness^37-41^. In particular, the effects on arterial stiffness of JUUL use were as yet unknown, although recent studies have shown that heart rate as well as mean arterial pressure are influenced by vaping the American JUUL version^42^. This is in accordance with our data which showed acute increases in peripheral and central blood pressure for a short period after both JUUL use and smoking.

Recently published studies provided a contradictory picture with regard to arterial stiffness after vaping and smoking^18,38,39,43^. In line with our previously published study on e-cigarettes, Franzen et al.^18^ and Vlachopoulos et al.^39^ showed an impact of vaping on central hemodynamic and peripheral blood pressure. As expected and already published^18,38,39,43^, heated tobacco products, cigarettes, and e-cigarettes with nicotine-containing liquids lead to an increase of peripheral and central hemodynamic parameters and arterial stiffness. For the JUUL device, we were also able to show an increase in peripheral and central hemodynamics that was comparable to the changes found with combustible cigarettes. With regard to these parameters, only minor differences were found between the new and old JUUL technology.

In line with the previously published trials, AIx@75 increased significantly after JUUL use. As another parameter of arterial stiffness, we showed significant raises in PWV in the combustible cigarette and JUUL groups. These temporary alterations of hemodynamic and arterial stiffness are likely triggered by nicotine as an acute vasoconstrictor and also by local and circulating catecholamines which are released during nicotine consumption. These catecholamines also indicate the activity and stimulation of sympathetic ganglia, among other things. As a consequence of the stimulation due to nicotine, sympathetic neuronal discharge-impaired nitric oxide production increases in the central nervous system, which in turn leads to an increase in arterial stiffness^44-46^. However, our results showed that a small part of the increase in arterial stiffness remained unexplained by the device, blood pressure, heart rate, or sex. This unexplained change might be due to the effects of the cigarette and JUUL on the endothelium. Therefore, a combination of nicotine and harmful or potentially harmful compounds in smoke and vapor is one of the most likely explanations for this observation. Similar results were shown in an animal model by Nabavizadeh et al.^47^ who compared combustible cigarettes with heated tobacco products. In addition, population-based studies on cardiovascular diseases found an association between e-cigarette use and increased risk of myocardial infarction^22^. However, the influence of former or current smoking is unclear. The pathogenesis of coronary heart disease and other cardiovascular events includes stiffening of the arteries^34,48^, an effect that could be shown here to occur with JUUL use. Besides central hemodynamics, arterial stiffness seems to be even more important than peripheral blood pressures as a surrogate parameter for cardiovascular events^30,49,50^. It should be noted that cigarette smoke not only affects the cardiovascular system but also has other negative effects on health, and e-cigarette aerosols contain much lower amounts of harmful and potentially harmful substances than cigarette smoke^10,25,51^. Thus, a complete switch from tobacco cigarettes to e-cigarettes is widely acknowledged to reduce the exposure to many carcinogens and other toxicologically relevant compounds^11,52^.

### Limitations

Unlike the study by Vlachopoulos et al.^39^ on blood pressure and arterial stiffness which included four study arms, the present study included only three arms. Also, the sample size was limited, and a cross-over design was not used. In addition, participants were not randomized to the study arms, and only acute effects were assessed. Last, the intensity of JUUL use, i.e. the intensity of the puff, could not be standardized, although the frequency, number, and duration of puffs were defined to avoid large differences in vaping behavior. In this study, other measurements like EEG and blood samples were carried out in parallel to increase the information output. However, possible influences on the individual stress level of the participants could not be excluded.

### Future research

Future studies should focus on the chronic effects of the JUUL device and compare them with the long-term effects of conventional e-cigarettes with nicotine-containing or nicotine-free liquids or combustible cigarettes. The literature describes neither endothelial dysfunction nor cardiac inflammation for JUUL use, so future studies should address these issues. Also, a head-to-head comparison of e-cigarettes, JUUL, heated tobacco products, and combustible cigarettes should be conducted and should focus on peripheral blood pressure, arterial stiffness, and endothelial dysfunction.

## CONCLUSIONS

Comparable adverse changes for blood pressure, heart rate, and arterial stiffness parameters were shown between both smoking a filter cigarette and JUUL use. In our interpretation, these and especially the changes in arterial stiffness may be associated with increased cardiovascular risk. However, a long-term follow-up evaluation of JUUL use and a head-to-head comparison with conventional e-cigarettes are still needed.

## Data Availability

The data supporting this research are available from the authors on reasonable request.
